# Challenges in the Interpretation of MRI Examinations Without Radiographic Correlation: Pearls and Pitfalls to Avoid

**DOI:** 10.7759/cureus.16419

**Published:** 2021-07-16

**Authors:** Ryan H Richter, Douglas Byerly, Donald Schultz, Liem T Mansfield

**Affiliations:** 1 Department of Radiology, Brooke Army Medical Center, San Antonio, USA; 2 Department of Radiology, Uniformed Services University of the Health Sciences, Bethesda, USA; 3 Department of Radiology, Wilford Hall Ambulatory Surgical Center, San Antonio, USA; 4 Radiology, Shannon Clinic, San Angelo, USA

**Keywords:** radiographs, mri, pearls, pitfalls, interpretation, infection, trauma, tumors, arthropathy

## Abstract

As physics introduces more complex and seemingly thorough techniques to evaluate patient symptoms, cross-sectional imaging, especially magnetic resonance imaging (MRI), seems like the modality of choice to best help patients. However, musculoskeletal radiology (MSK) requires not just the excellent soft-tissue contrast provided by MRI but also an evaluation of the aggressiveness of a lesion, a detailed evaluation of osseous anatomy or distribution of disease, and a way to easily identify calcifications and gas in soft tissue in order to make the correct diagnosis. This article will demonstrate, through numerous cases, the importance of radiographs in the full characterization of MSK-related pathology. It will focus on imaging pearls and pitfalls to avoid when radiographs are not available and discuss the findings that can be expected if comparison radiographs were available.

## Introduction and background

Magnetic resonance imaging (MRI) has become increasingly prominent in modern medicine. To put this into perspective, consider that 40.44 million MRI examinations were performed in the United States in 2019 [1]. Important to note is that it is not uncommon for radiologists to be faced with the interpretation of these MRI examinations without radiographic correlation despite the guidelines provided by the American College of Radiology (ACR) appropriateness criteria. The reasons for the lack of radiographic correlation are unclear and likely multifactorial; however, radiographs have been shown to be essential to MRI interpretation in 61%-75% of cases in one study [2].

The strengths of MRI imaging are excellent soft-tissue contrast resolution, multiplanar imaging, and lack of ionizing radiation. However, a common pitfall when using MRI to evaluate a mass is the mischaracterization of the aggressiveness of a lesion. Some benign lesions, such as chondroblastoma or infection, can have an aggressive appearance on MRI, causing the radiologist to consider a malignant process. Likewise, an aggressive malignant lesion can have well-defined margins on MRI, mimicking a less aggressive process. A pearl is that aggressive appearance on imaging does not equate to malignancy and while MRI better assesses the extent of involvement of a lesion, radiographs are often better or at least complementary for the characterization of aggressiveness. Remember that the tumor matrix (chondroid or osteoid) is often better assessed on radiographs and is a key feature in narrowing the differential diagnosis. Additionally, radiographs are important in evaluating the surrounding osseous anatomy, including the distribution of disease, and in evaluating the presence of calcifications and gas within the soft tissue. Because of this, the lack of comparison radiographs may lead to misinterpretation, additional/unnecessary imaging, or unnecessary treatments.

This article will evaluate different conditions in which radiographs are essential to the interpretation of MRI examinations, focusing on potential pitfalls and highlighting diagnostic pearls. These conditions will be grouped into four broad entities: arthropathy/deposition disease; infection; trauma; and tumors (both soft tissue and osseous).

## Review

Arthropathy/deposition disease

Calcium Hydroxyapatite Deposition Disease (HADD)

HADD manifests by the deposition of periarticular calcifications most commonly affecting the shoulder; however, it can involve other joints such as the hips near the greater trochanter, the wrist, or involving the digits. HADD can be exquisitely painful or asymptomatic and can present with a wide spectrum of imaging features, causing a diagnostic conundrum when interpreted without radiographs.

In the inflammatory phase, the calcifications may be surrounded by robust soft tissue edema (T2 hyperintense and T1 hypointense signal) in and around tendons such as the rotator cuff tendons, bursar fluid collections, such as the subacromial-subdeltoid bursa, intra-articular rupture resulting in a destructive inflammatory arthropathy termed Milwaukee shoulder, or with intraosseous involvement where HADD has the ability to incite bone marrow edema. All of these findings can mimic other processes such as other inflammatory arthropathies (rheumatoid arthritis (RA), gout or calcium pyrophosphate deposition disease (CPPD)), infection (septic arthritis, osteomyelitis, or infected bursitis), or neoplasm.

**Figure 1:**
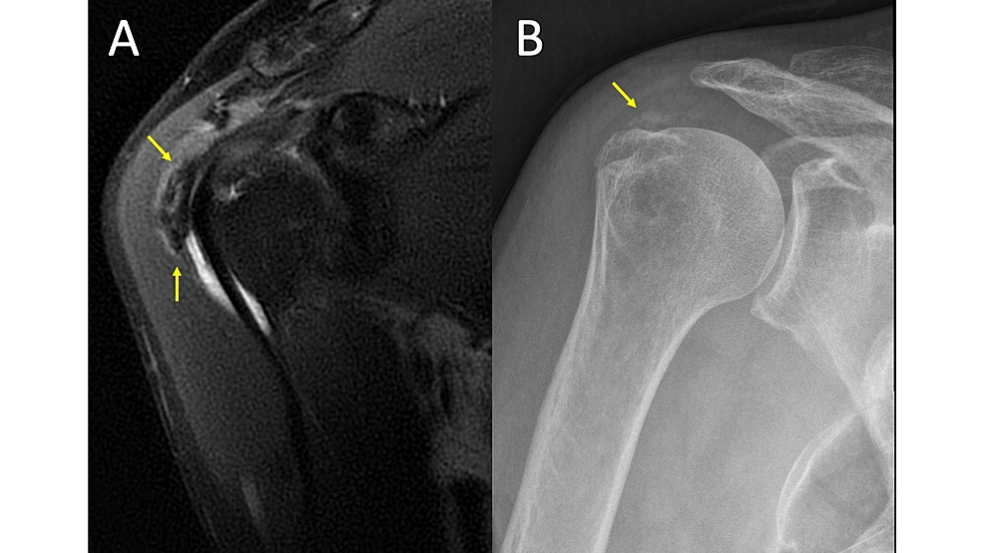
Hydroxyapatite Deposition Disease MRI coronal T2 fat saturation (FS) (1A) and plain radiograph of the shoulder in the Grashey view (1B) demonstrate mineralization within the soft tissues adjacent to the greater tuberosity (arrows). While easy to appreciate on the MRI in this case because of the extent of calcification and interval rupture into the bursa (MRI obtained several months after the radiographs), HADD can be difficult to distinguish from surrounding tendons and easily overlooked on MRI without comparison radiographs.

In the quiescent phase, HADD is typically hypointense on T1, T2, and PD weighted sequences or imaging and can be difficult to detect when adjacent to other hypointense structures such as tendons, ligaments, or cortical bone (Figure 1, panel A). A useful mnemonic for T2 hypointense lesions is CHAFTS (Calcium, Hemosiderin, Amyloid or Air, Fibrosis, Tophi, and Synovitis) [3]. Mineralization related to HADD is much more obvious on radiographs that are diagnostic when their classic cloud-like morphology without cortication or trabeculations are seen (Figure 1, panel B). Careful evaluation in periarticular locations is required, as these findings can be faint in appearance or overlie bone on some views. When radiographs are not available, soft tissue mineralization is best appreciated on T1 sequences, as it will be more hypointense than the surrounding tendons and bursae while often blending with these structures on T2 sequences.

Rheumatoid Arthritis (RA)

There is no question that MRI is superior to radiographs in the early detection of RA, as it recognizes early synovial inflammation before osseous changes occur. This is especially helpful when the typical symptoms of early morning stiffness, pain, and swelling are present [4]. However, RA findings can be more obscure when presenting with atypical symptoms or occurring in an atypical joint.

**Figure 2:**
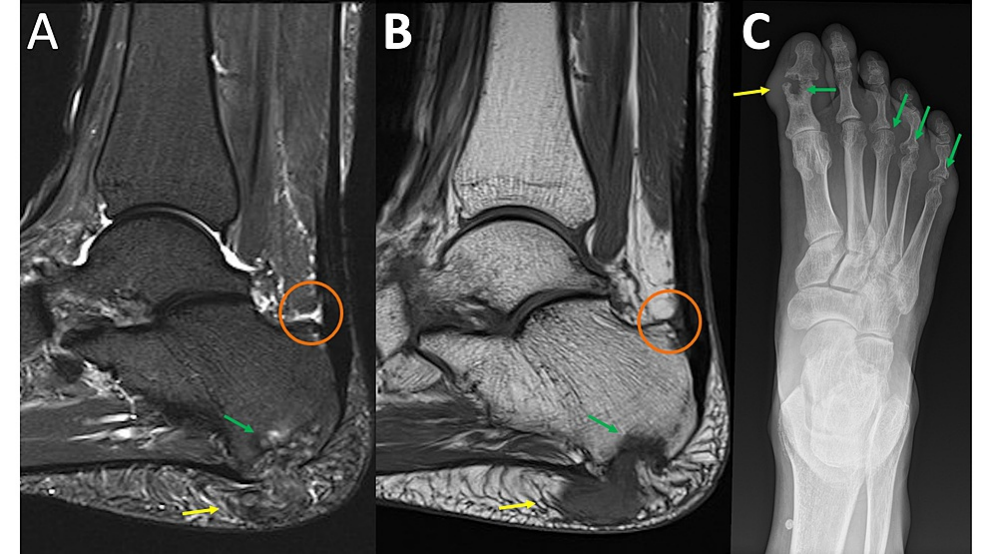
Rheumatoid Arthritis MRI sagittal T2 fat saturation (FS) (2A) and MRI sagittal T1 (2B) demonstrate a heterogenous predominately intermediate signal intensity mass within the subcutaneous soft tissues of the heel fat pad (yellow arrows) with extension/erosion of the adjacent calcaneus (green arrows). Upon close inspection, there is a T2 hyperintense signal deep to the calcaneal insertion of the Achilles tendon (circle). The diagnosis becomes much clearer when the comparison radiograph of the foot is reviewed (2C) demonstrating extensive erosive changes to the first interphalangeal joint and the 3rd-5th metatarsal heads (green arrows). In addition, there is soft tissue prominence along the first interphalangeal joint likely representing an additional site of an additional rheumatoid nodule (yellow arrow).

Without a clinical history of RA and without small joint comparison radiographs, a soft tissue mass with intermediate heterogeneous signal in the subcutaneous soft tissue with extension/erosion into the adjacent calcaneus may be mistaken for soft tissue malignancy (Figure 2, panels A-B). Comparison radiographs showing small joint erosions in a symmetric distribution without periostitis, enthesopathy, or ankyloses helps one recognize that the soft tissue mass is likely a rheumatoid nodule (Figure 2, panel C).

Remember that osseous erosions adjacent to the soft tissue mass, patient demographics, multifocal process, and review of records for the involvement of classic joints may simplify the diagnosis.

Gout

Similar to RA, atypical symptoms occurring in an atypical joint or initial presentation early in the patient’s disease process when radiographs are often negative may lead to a request for advanced imaging.

**Figure 3:**
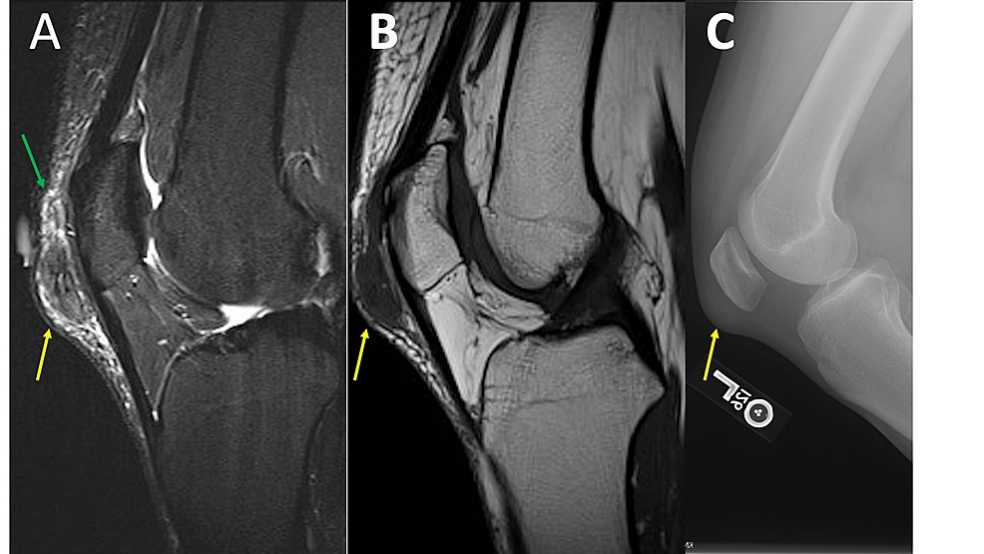
Gout MRI sagittal T2 fat saturation (FS) (3A) and MRI sagittal T1 (3B) demonstrate a nonspecific heterogeneous, predominately intermediate signal intensity soft tissue mass (yellow arrow) with surrounding soft tissue edema (green arrow) superficial to the patella and patellar tendon. Lateral knee radiograph (3C) demonstrates an area of soft tissue prominence (yellow arrow) corresponding to the mass noted on MRI. The mass appears slightly dense but there are no associated mineralized bodies, which would be a helpful discriminator. Also, no erosive changes are present. It is also important to recognize that the patella and patellar tendon are common sites for tophus formation. A search of the patient’s radiology records demonstrated findings suggestive of gout involving the foot (not shown).

The MRI features of gout are highly variable. Tophi have an intermediate to low signal on T1-weighted imaging and heterogeneous signal on fluid sensitive sequences (Figure 3, panels A-B). They can enhance uniformly or have non-enhancing centers. Bone erosions adjacent to tophi can produce cortical disruption and a variable degree of bone marrow edema [5]. Tophi and large erosions of less common locations and in younger patients can be mistaken as osteomyelitis or malignancy [5]. Radiographs can be used to evaluate for juxta-articular erosion with sclerotic margin, an overhanging edge, and/or amorphous calcifications associated with soft tissue mass (tophi) to support the diagnosis of gout (Figure 3, panel C). As previously mentioned, a review of the patient’s medical records for radiographs of more commonly involved joints can be helpful.

Also remember that malignant intra-articular processes are rare. Intra-articular masses can be categorized as synovial (RA, lipoma arborescence, or pigmented villonodular synovitis), deposition disease (gout or amyloid), infectious, vascular (synovial hemangioma), malignant (metastasis, synovial sarcoma, or chondrosarcoma), and post-surgical (arthrofibrosis (aka cyclops lesion)). A patient’s demographics and history can help order the differential diagnosis.

Neuropathic Arthropathy

Presenting with a swollen unstable joint, neuropathic (Charcot) arthropathy is a rapidly progressive and severely destructive process. MRI aids in problem-solving the cause of osseous destruction and can be particularly helpful in differentiating between neuropathic arthropathy and osteomyelitis. However, anatomy can be severely distorted and difficult to interpret on MRI. Radiographs allow for easier assessment of osseous anatomy [6]. Diagnosis is made when the 5 Ds are seen: Distended joint (effusion), Dislocation, Debris (osseous), Disorganization, and Density of bone increased [7].

*Hemophilia* 

Like many of the disease processes already discussed, the MRI appearance can be aggressive and nonspecific, mimicking other processes. The classic radiographic features are radiodense soft tissue swelling and effusions; osteopenia (juxta-articular or diffuse); epiphyseal overgrowth (widened intercondylar notch when the knee is involved); and subchondral cysts [8]. Hemophilic arthropathy can be a mono- or polyarticular process with an asymmetric sporadic distribution. The knee, elbow, ankle, and hip (in decreasing order of prevalence) are most commonly affected and are often more easily recognized. MRI features are non-specific, demonstrating thickened synovium with hypointense signal due to hemosiderin deposition, which is best appreciated on gradient echo sequences (GRE) sequences because of blooming artifact, cartilage loss, and erosions. MRI may also be confusing, as the classic radiographic appearances, such as epiphyseal over-growth, is generally presented on radiographs in an educational setting. Radiologists and clinicians should be able to identify classic radiograph findings on advanced imaging. Like other processes, the demographics and history are extremely helpful, as this is an X-linked recessive bleeding disorder that occurs almost exclusively in males. Roughly 50% of hemophilia patients will develop severe arthropathy.

Tumoral Calcinosis

**Figure 4:**
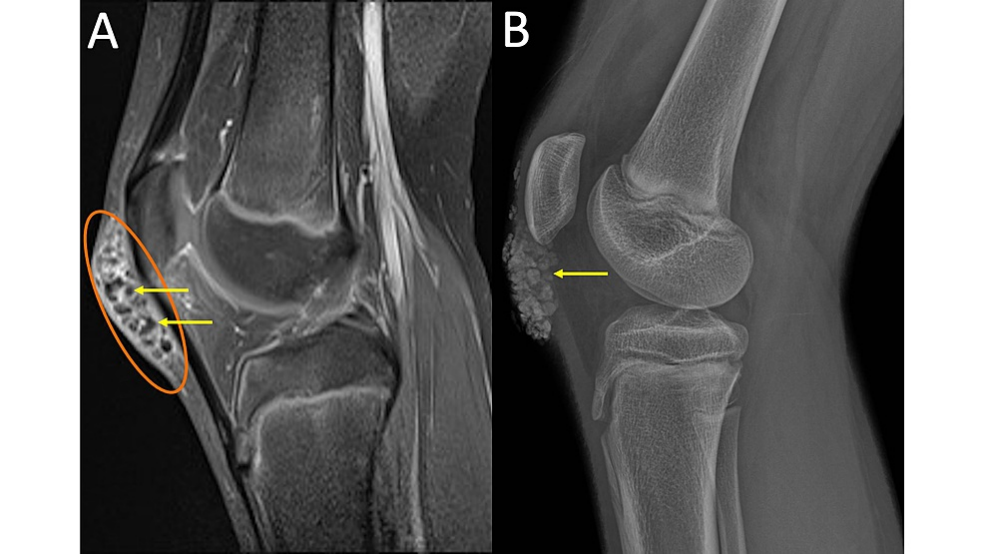
Tumoral Calcinosis MRI sagittal T1 post-contrast fat saturation (FS) of the right knee (4A) demonstrates multiple T1 hypointense rounded structures (yellow arrows) with intervening regions of enhancing synovium (orange oval) anterior to the patella and patellar tendon within the region of the prepatellar bursa. The right knee radiograph (4B) allows for easy recognition that the previously described T1 hypointense structures correspond to coarse lobulated calcifications (yellow arrow). There is no scalloping of adjacent bone nor other aggressive osseous features.

Initially presenting as an enlarged painless joint, tumoral calcinosis is most commonly due to renal failure or hereditary disorders in phosphate regulation. This leads to profuse soft tissue calcifications, which occasionally liquefy to form milk of calcium. On MRI, T1 heterogeneously hypointense signal and T2 hyperintense cysts with low signal sediment and surrounding edema can be mistaken for other causes of metabolic or dystrophic calcifications (Figure 4, panel A). As previously mentioned, calcifications are often more readily identified on radiographs and easily overlooked on MRI (Figure 4, panel B). Radiographs will show cloudlike calcifications in a periarticular distribution without destructive osseous findings, confirming the diagnosis. A history of renal failure or other metabolic derangements is seldom provided with the order for musculoskeletal examinations and, therefore, it is often helpful to review the patient’s imaging records for other signs of metabolic derangement or renal failure such as atrophied kidneys, dialysis catheters, peripheral vascular disease, or additional locations of soft tissue calcifications [9].

Scleroderma

Scleroderma is a multisystem autoimmune connective tissue disorder with variable presentation. Musculoskeletal manifestations can involve both osseous structures and soft tissues. MRI is nonspecific and may be confused for trauma, infection, soft tissue neoplasm, tenosynovitis, other arthropathies, and/or myopathy. Radiographs are diagnostic when showing acro-osteolysis and soft tissue calcifications. Additionally, acro-osteolysis at the phalangeal tufts with osseous resorption of the first carpometacarpal joint resulting in radial subluxation is specific to scleroderma.

Calcium Pyrophosphate Deposition Disease (CPPD)

CPPD arthropathy is the most common crystal deposition arthropathy typically presenting in middle-aged or elderly patients. It can be asymptomatic or mimic symptoms of gout, RA, osteoarthritis (OA), and neuropathic arthropathy. Chondrocalcinosis is nonspecific and reported in 5% of the population with increased prevalence with age [10]. When present and in the appropriate setting, it can help distinguish CPPD arthropathy from other processes [11].

**Figure 5:**
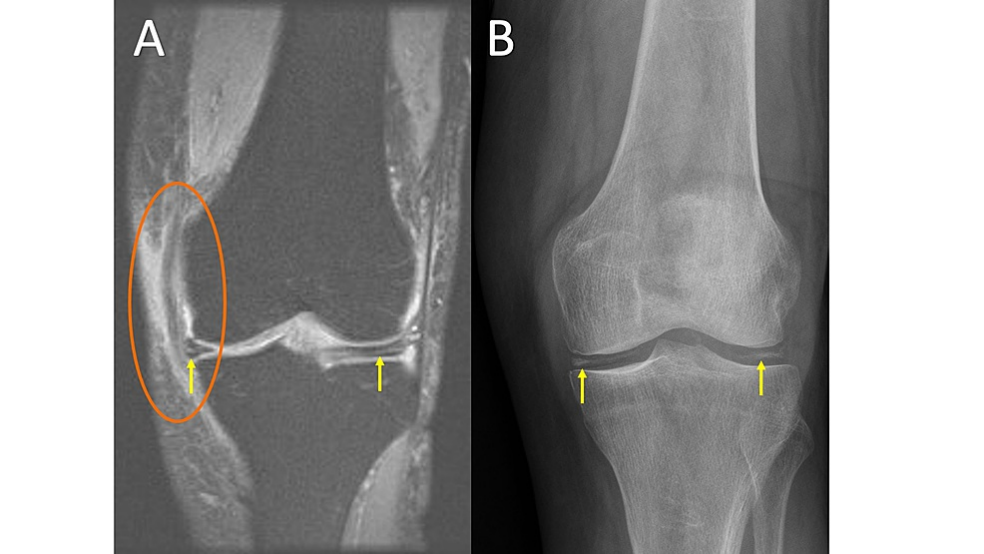
Calcium Pyrophosphate Deposition Disease MRI coronal T2 fat saturation (FS) (5A) demonstrates a linear hyperintense T2 signal within the menisci (arrows). Incidentally, the medial collateral ligament is thickened and heterogenous with increased T2 signal both within and around the ligament consistent with a sprain (circle). Anteroposterior (AP) knee radiograph (5B) demonstrates chondrocalcinosis of the menisci (arrows).

Chondrocalcinosis is difficult to appreciate on MRI (Figure 5, panel A); however, the faint punctate and linear calcifications deposited in hyaline and fibrocartilage (labrum, TFCC, and meniscus) are easily identified on radiographs, as demonstrated in Figure 5, panel B [12]. The presence of chondrocalcinosis in the setting of erosive arthropathy points to the diagnosis of CPPD arthropathy. Other clues include uniform joint space loss, osteophyte formation (not typical of other erosive arthropathies), and bilateral distribution. Commonly involved joints include the knees, hands, hips, and pubic symphysis in decreasing order of prevalence. When the knees are involved, there is uniform joint space loss predominately affecting the patellofemoral compartment. Classic imaging features of hand/wrist involvement include chondrocalcinosis of the scapholunate ligament and triangular fibrocartilage, hook-like osteophytes of the second and third metacarpal heads, and in advanced cases, disruption of the scapholunate ligament with proximal migration of the capitate, and scapholunate advanced collapse (SLAC wrist).

A pitfall associated with chondrocalcinosis on MRI imaging is related to its high T2 signal within fibrocartilage. The high T2 signal from chondrocalcinosis within menisci, labrum, or TFCC can be mistaken for tears, especially when comparison radiographs are not available for review at the time of MRI interpretation.

Infection

Septic Arthritis

Traditionally, septic arthritis presents as a painful, warm, and swollen joint with decreased range of motion. When clinical suspicion is high, joint aspiration should be performed early, as it is diagnostic. This allows for rapid initiation of treatment (improving outcome) and antibiotics can be tailored from the cultures. In less straightforward cases, radiographs and MRI are obtained though MRI does not have specific findings early in the disease [13].

**Figure 6:**
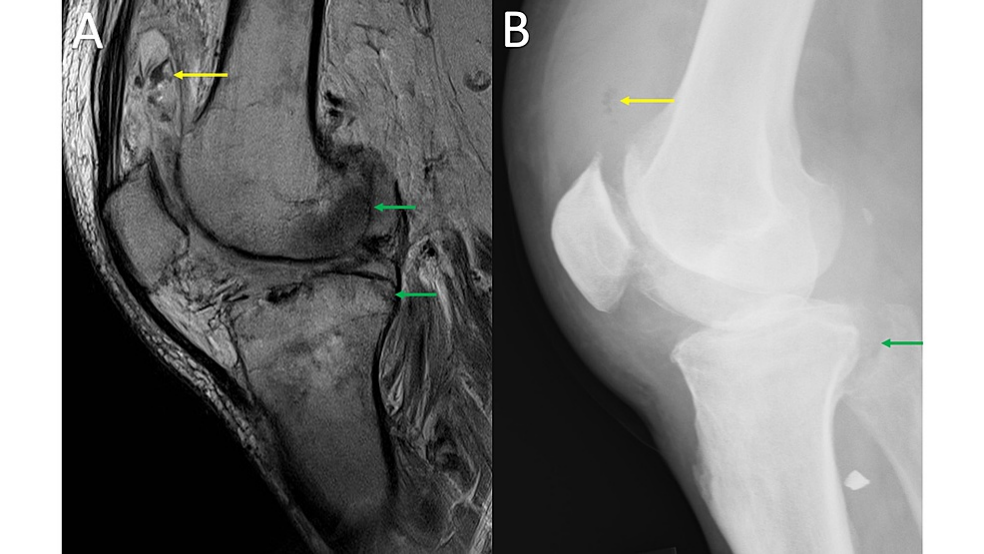
Septic Arthritis MRI sagittal proton density (PD) of the right knee (6A) shows thickened edematous synovium, subcutaneous edema, and large suprapatellar joint effusion with low PD signal (yellow arrow) within the suprapatellar bursa. Lateral radiograph of the right knee (6B) allows for easy recognition of gas (yellow arrow) within the suprapatellar bursa. Of note, there is also an abnormal bone marrow signal in the distal femur and proximal tibia (green arrows) with a radiograph showing cortical destruction involving the posterior tibia adjacent to the proximal tib-fib joint (green arrow). The final diagnosis was septic arthritis and osteomyelitis.

The first radiographic sign of septic arthritis is a joint effusion; however, this is also nonspecific. The most important radiographically visible sign of infection is the identification of gas. On MRI, gas will appear hypointense on both T2 and T1, like calcifications, and can easily be overlooked, blending with tendons, fibrocartilage, ligaments, or cortex (Figure 6, panel A). The lucency associated with soft tissue gas is more readily appreciated on radiographs (Figure 6, panel B). It is important to remember that other potential sources of gas may be secondary to a recent procedure such as joint injection/aspiration.

Necrotizing Fasciitis

Presenting with severe pain, swelling, and crepitus, necrotizing fasciitis is most commonly caused by a polymicrobial process. MRI is nonspecific, showing soft tissue edema and thickened, enhancing fascia with increased T2 signal [14]. As previously discussed, gas can be easily overlooked on MRI, appearing as small foci of low signal on both T1 and T2 and is more readily appreciated as small round or oval lucent foci on radiographs, which are more easily and rapidly obtained. Necrotizing fasciitis is generally diagnosed clinically and requires urgent surgical debridement. In many cases, MRI is unnecessary and may lead to a delay in treatment [15].

Osteomyelitis

**Figure 7:**
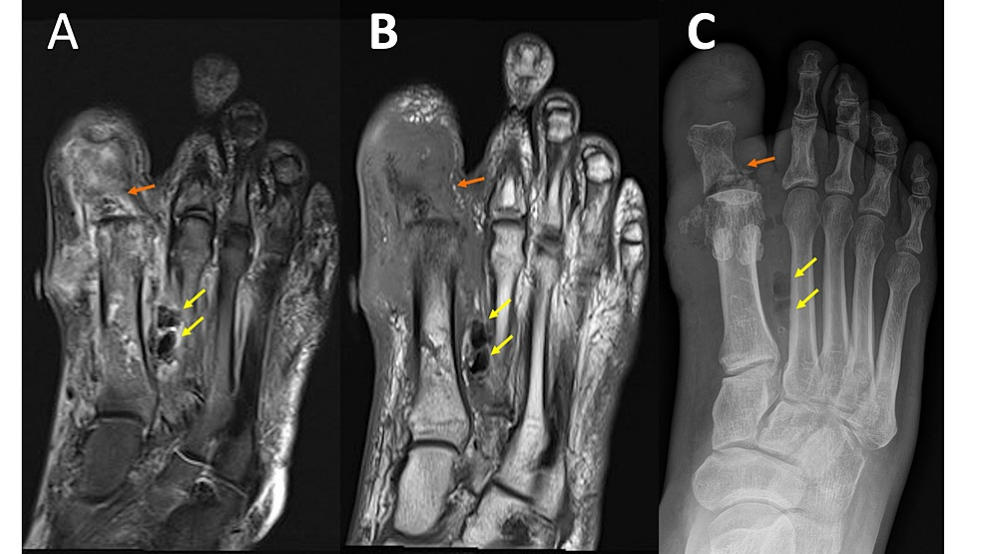
Osteomyelitis MRI coronal T2 fat saturation (FS) (7A) and MRI coronal T1 (7B) demonstrates marrow edema predominately in the first metatarsal and phalanges with surrounding soft tissue edema and areas of hypointense T1/T2 signal between the first and second metatarsals (yellow arrows). Extensive/near-complete destruction of the first distal phalanx and medial margin of the first metatarsal head with associated soft tissue mineralized debris (orange arrows). The mineralized debris is not well-appreciated on the MRI images. A marker is noted along the medial foot, marking an ulcer appreciated on the exam but not well-displayed on the provided images. Edema extends from the ulcer to the underlying first metatarsal. Left foot radiograph (7C) illustrates extensive destruction of the first distal phalanx, the base of the first proximal phalanx (orange arrow), and medial margin of the first metatarsal head with associated soft tissue mineralized debris. Hypointense T1/T2 gas is much easier to appreciate on the radiograph (yellow arrows).

Similar to RA, MRI is more sensitive than radiographs in the early detection of osteomyelitis, showing noticeably low T1 and high T2 signals within the bone marrow. Other imaging features of osteomyelitis include periostitis and cortical destruction [16]. Radiographs are a useful first step because they are readily available, quick to obtain, and inexpensive. A pitfall of radiographs is that they may be negative early in the process. As previously discussed, gas associated with infection may be more obvious on radiographs, and the overall osseous anatomy in advanced destructive osteomyelitis is better appreciated (see discussion on highly destructive processes in the section titled neuropathic arthropathy). These findings are exemplified in Figure 7, panels A-C. In addition to high sensitivity and early detection, MRI allows the evaluation of the extent of soft tissue involvement (cellulitis, abscess formation, and fasciitis), as well as the extent of osseous and joint involvement, which is generally underestimated on radiographs and physical exams, thereby aiding in management planning. Osteomyelitis in adults is most commonly seen in the setting of direct extension from a penetrating injury or chronic ulceration. It is helpful if these areas are marked by the MRI technologist prior to imaging. Soft tissue defects or foreign bodies can sometimes be appreciated on radiographs more easily than MRI, however, markers are also helpful for radiographic assessment.

Without the typical presentation of pain, decreased range of motion, trouble bearing weight, swelling, and fever, MRI findings can be similar to findings seen with trauma, tumor, neuropathic arthropathy, and advanced erosive arthropathy. In these cases, history, laboratory values, and distribution are important factors for establishing the diagnosis. A review of the patient’s radiology records may sometimes reveal a pertinent history, such as bilateral foot or hand radiographs due to a prior workup of inflammatory arthropathy, or advanced atherosclerosis as can be seen in chronic diabetes/peripheral neuropathy suggesting neuropathic arthropathy. In some cases, especially when there are superimposed etiologies, tissue sampling may be indicated. It should be noted that in the setting of osteomyelitis, bone cultures are not uncommonly negative and do not exclude infection. In these situations, tissue sampling should include the adjacent soft tissues and periosteum when a bone biopsy is performed [17].

Trauma

Myositis Ossificans

**Figure 8:**
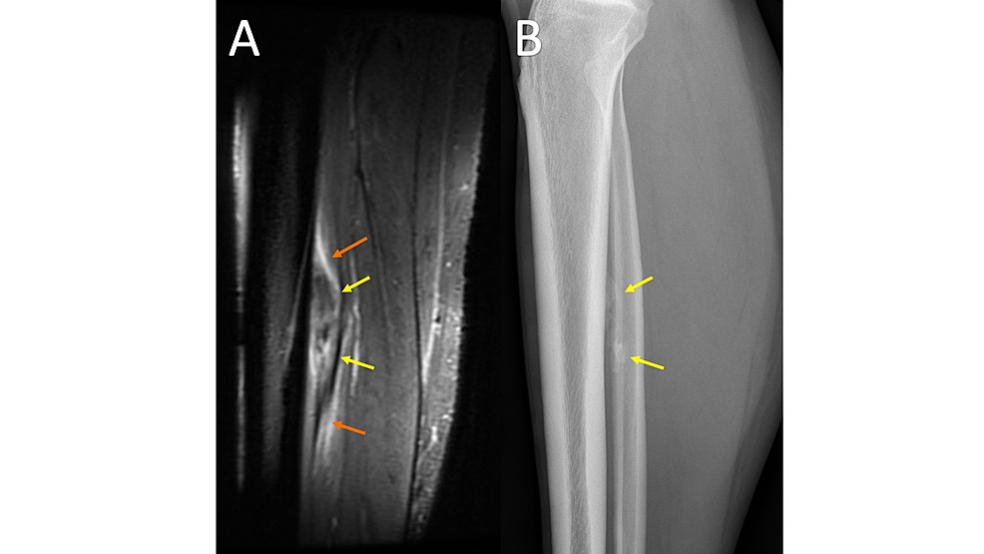
Myositis Ossificans MRI sagittal T2 fat saturation (FS) (8A) obtained at the time of initial presentation demonstrates an ill-defined T2 hypointense region (yellow arrows) with a surrounding T2 hyperintense signal likely representing edema (orange arrows), a non-specific imaging appearance. Lateral tibia/fibula radiograph (8B) shows immature mineralization (yellow arrows), which, especially with the clinical history, aids in the diagnosis of myositis ossificans. A follow-up radiograph six weeks later (not shown) demonstrated the development of peripheral mineralization with central lucency consistent with myositis ossificans.

Myositis ossificans (MO) typically presents as a soft tissue mass in the setting of trauma but is also associated with burns and neurologic disorders. However, in many cases, the patient may not remember the inciting factor and advanced imaging may be obtained to evaluate a palpable soft tissue mass. The MRI appearance of MO varies with age. Early features of MO include a T1 hypointense-to-intermediate mass, with surrounding T2 hyperintense reactive edema lasting up to eight weeks with variable heterogeneous intermediate to high T2 signals centrally depending on the extent of cellularity and cartilage components (Figure 8, panel A). In mature MO, there will be a peripheral T1/T2 hypointense rim corresponding to mature bone, while centrally, it will be a heterogeneous intermediate to high T1 and T2 signal [18]. If close to the bone, there may be reactive bone marrow edema and periosteal reaction. In some cases, when close to the bone, it can be mistaken for a surface lesion (periosteal or juxtacortical).

MO can be misdiagnosed as a neoplasm on MRI, especially early in the maturation process when the lesion has a nonspecific appearance. When MO is a clinical consideration for a soft tissue mass, it is critical to perform short interval follow-up imaging and a clinical exam and obtain radiographs to assess resolution or normal maturation (Figure 8, panel B). On follow-up imaging, myositis ossificans will demonstrate the development of peripheral mineralization with central lucency on radiographs, which is a pathognomic finding.

Calcific Myonecrosis 

**Figure 9:**
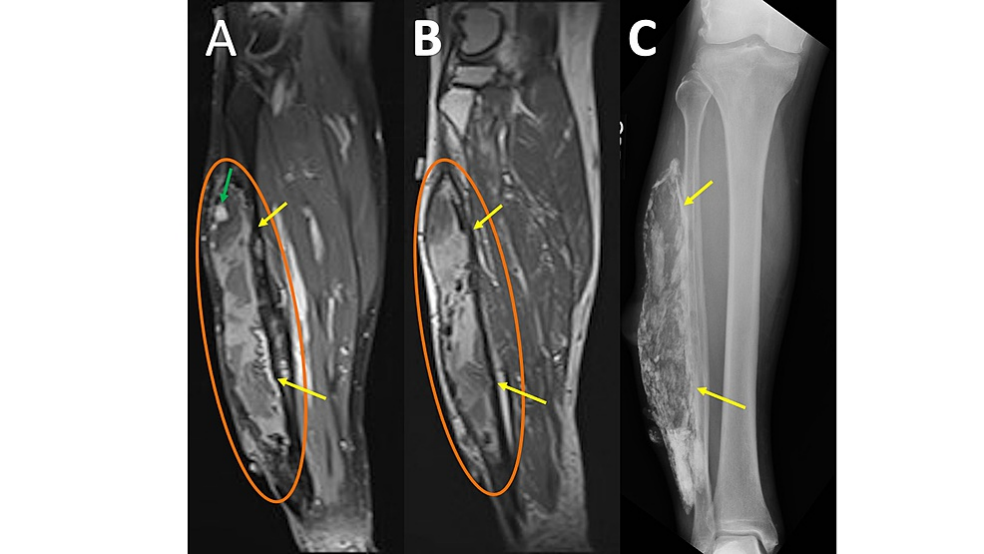
Calcified Myonecrosis MRI coronal T1 fat saturation (FS) post-contrast (9A) and MRI coronal T1 (9B) show a heterogeneous mass (oval) with scattered foci of internal enhancement (green arrow) replacing the majority of the peroneus longus and brevis muscles. Also noted is a peripheral T1/T2 hypointense capsule (yellow arrows). The extensive peripheral and internal soft tissue calcifications are better appreciated on the comparison tibia/fibula radiograph (9C).

Calcific myonecrosis is characterized by a painful, slowly enlarging, soft tissue mass. Unlike myositis ossificans, the inciting factor is usually remembered by the patient and can include a history of compartment syndrome or vascular or neurologic injury. MRI shows a well-circumscribed mass with a heterogeneous signal on T2 (Figure 9, panel A). T1 shows a homogenous intermediate signal throughout the central fluid region (Figure 9, panel B) with a corresponding T2 hyperintense signal [19]. The heterogeneous appearance of the muscle with an area of enhancement can be mistaken as a tumor (e.g. sarcoma) when the capsule is thin and thus easily overlooked. Radiographs show a fusiform mass with peripherally oriented, plaque-like, amorphous calcifications aiding in diagnosis, especially when calcifications correspond to the surrounding capsule (Figure 9, panel C).

Ossicles/Sesamoids (Displaced or Fractured)

**Figure 10:**
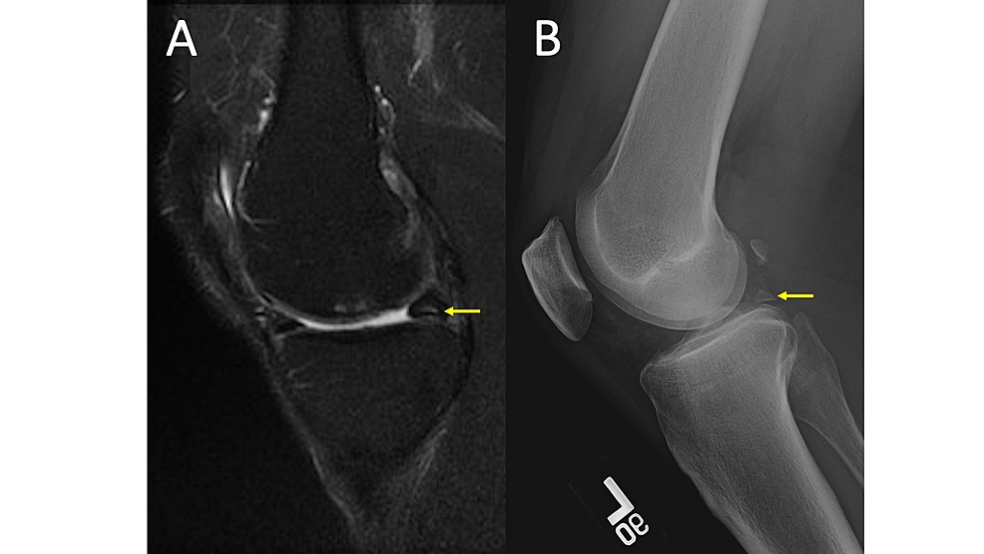
Meniscal Ossicle MRI coronal T2 fat saturation (FS) (10A) shows intermediate to hyperintense T2 signal noted within the posterior horn of the medial meniscus (yellow arrow), which may be interpreted as a meniscal tear or intrasubstance degeneration. A lateral knee radiograph (10B) demonstrates an ossified triangular-shaped body within the posterior joint space (yellow arrow) revealing the apparent meniscal tear or intrasubstance degeneration to be a meniscal ossicle.

Meniscal ossicles may be difficult to identify on MRI examinations if the ossicle is highly mineralized and the MRI protocol only has fat-saturated sequences, as the ossicle may be isointense to the surrounding meniscus. If the ossicle is not highly mineralized and instead demonstrates a T2 hyperintense signal, it may be confused for a meniscal tear, intra-articular body, or intrameniscal cyst (Figure 10, panel A). Meniscal ossicles are easily identified on radiographs, classically as an ovoid or triangular, corticated osseous body (Figure 10, panel B), most commonly in the posterior horn of the medial meniscus [20]. Its triangular shape in the appropriate location helps distinguish it from an intra-articular body or “ossicle such as a fabella.”

Once a meniscal ossicle has been identified, a close scrutinization of the posterior root ligaments of the corresponding meniscus is recommended, as meniscal ossicles are commonly associated with root tears [21]. Also look for secondary signs of meniscus root tear, such as meniscal extrusion, which is best appreciated on the coronal images.

In addition to meniscal ossicles, sesamoids/ossicles can also be located within tendons, ligaments, or periarticular tissues. When present within tendons, they present as focal areas of ossification, as the tendon passes over joints or bones. Similar to a meniscal ossicle, MRI appearance follows the bone on all sequences. These also can be difficult to appreciate on fat-suppressed sequences due to blending with surrounding structures.

When fractured or displaced, sesamoids/ossicles can be mistaken as soft-tissue tumors, intra-articular bodies, or soft tissue injuries, especially when there is significant surrounding soft tissue edema. Not surprisingly, ossified ossicles and ossicle fractures are more readily appreciated on radiographs. When an ossicle is displaced, there should be a high suspicion of an associated tendon rupture. Similarly, small ligament avulsion injuries are better visualized on radiographs.

A common pitfall is overlooking subtle small areas of bone marrow edema and tiny avulsion injuries associated with a second fracture or soft tissue injury (ligamentous avulsion) on MRI, whereas the osseous fragment is easily seen on radiographs. It should be noted that avulsion fractures are well-known to elicit relatively minor, small areas of bone marrow edema in comparison to bone contusion or impaction injuries [22].

Tumors (osseous and soft tissue)

Vascular Malformations

**Figure 11:**
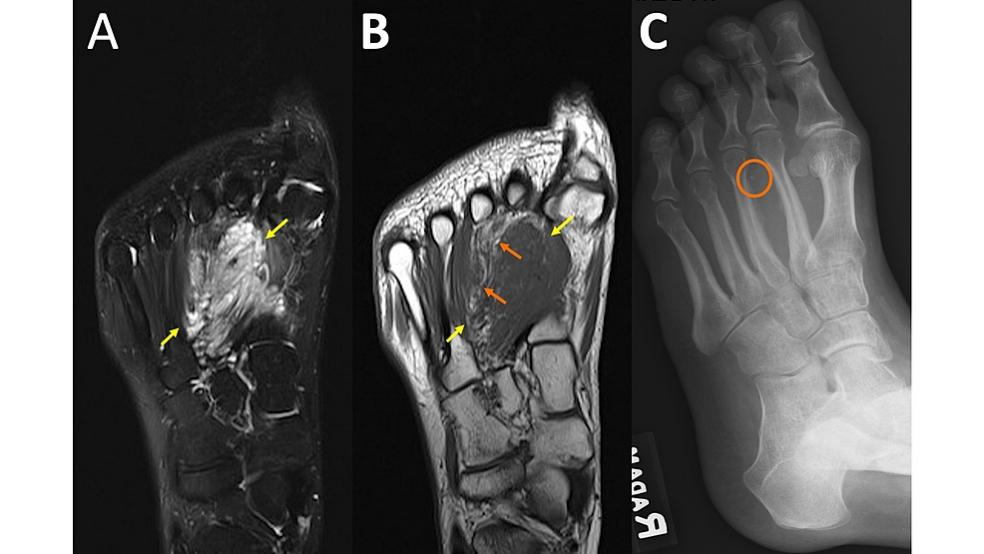
Vascular Malformation MRI long-axis T2 fat saturation (FS) (11A) and MRI long-axis T1 (11B) demonstrate a heterogenous predominately T2 hyperintense, T1 hypointense, lobulated, somewhat infiltrating mass (yellow arrows). There are some areas of T1 hyperintense signal suggesting intralesional fat (orange arrows), which is helpful for forming a differential diagnosis; however, the MRI is nonspecific. Considerations, in this case, would include but are not limited to a Desmond tumor, vascular malformation, or synovial sarcoma. Oblique foot radiograph (11C) demonstrates mild splaying of the second and third metatarsal bones with the chronic remodeling of the third metatarsal diaphysis, and the most important finding, soft tissue calcification consistent with a phlebolith (circle). This key feature helps establish the diagnosis of hemangioma, precluding the need for biopsy.

Hemangiomas and other vascular malformations of the soft tissue present as a slow-growing mass with additional characteristics depending on the subtype. MRI appearance is variable but includes areas of T1 hyperintensity corresponding to fat and a slow-flow mass that is T2 hyperintense to muscle and contrast enhancement of vascular portions (Figure 11, panels A-B). The variable appearance can sometimes be nonspecific and confusing on MRI especially when the lesion has little to no internal fat. In these cases, considerations would also include, but are not limited to, desmoid, synovial sarcoma, or other myxoid tumors. Radiographs showing phleboliths (Figure 11, panel C) together with the MRI findings are consistent with a vascular malformation, alleviating the need for tissue sampling [23]. Remember, as previously discussed, soft tissue calcifications are more obvious on radiographs; therefore, when considering hemangioma or vascular malformation in the differential diagnosis on cross-sectional imaging, look at the radiographs for the presence of phleboliths [24]. If no radiographs are available, phleboliths may be appreciated on the T1 sequences. If still not identified, recommend them prior to tissue sampling.

Synovial/Tenosynovial Chondromatosis

**Figure 12:**
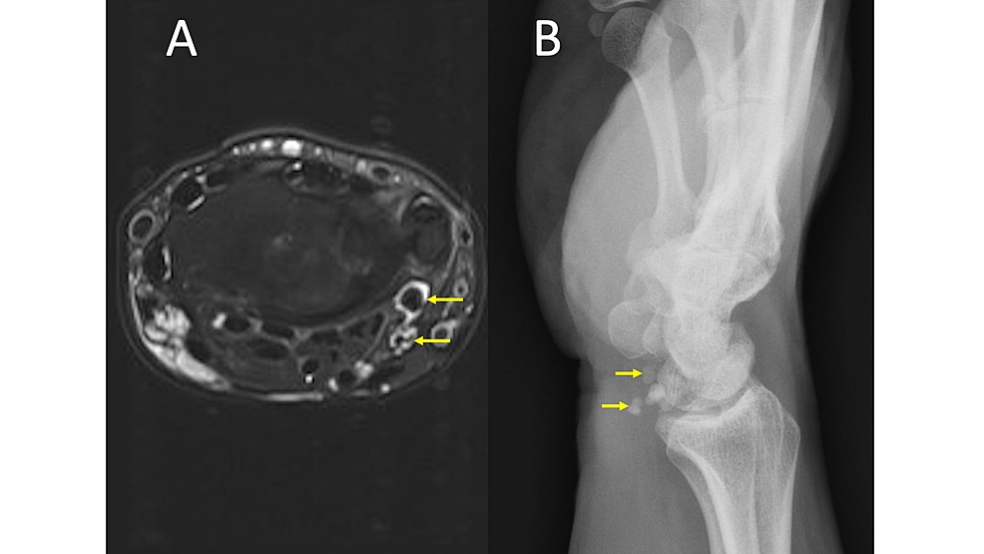
Synovial Osteochondromatosis MRI axial T2 fat saturation (FS) (12A) shows multiple small, round, hypointense, similar-sized foci along the volar wrist on both the radial and ulnar sides surrounded by fluid (yellow arrows). Lateral radiograph of the wrist (12B) demonstrates multiple mineralized bodies along the volar aspect of the wrist (yellow arrows).

Synovial or tenosynovial osteochondromatosis can be primary (neoplastic), where the bodies are similar in size, or secondary (mechanical injury) in the setting of degenerative osteoarthritis, where the bodies are generally more variable in size [25]. Osteochondromatosis may be difficult to appreciate on MRI alone, especially in small joints, such as the wrist (Figure 12, panel A), where there are several hypointense structures, such as tendons, ligaments, cortical bone, or vascular flow voids (don’t forget CHAFTS), which may obscure small adjacent ossified or cartilaginous bodies. Additionally, the differential diagnosis may be broader without radiographs and could include septic arthritis, inflammatory arthropathies, such as gout or RA, nodular synovitis, or neoplasms such as giant cell tumor of the tendon sheath, if they are located in the tenosynovial sheath.

Radiographs readily identify the characteristic multiple intra-articular ossified bodies distributed evenly throughout the joint in osteochondromatosis as shown in Figure 12, panel B [25]. Be sure to always scrutinize T2 hypointense structures surrounded by fluid and/or edema for the presence of bodies. Additionally, assess T2 hypointense structures in two orthogonal planes to distinguish between tendons or ligaments and round mineralized foci.

Osteoid Osteoma

Osteoid osteoma is a benign tumor classically presenting as aching pain, worse at night, and relieved by nonsteroidal anti-inflammatory drugs (NSAIDs). Central nidus is T1 hypointense with a variable signal on T2. There is surrounding bone marrow edema and enhancement of the nidus, which can make it difficult to differentiate from other conditions such as infection, inflammatory/non-inflammatory arthritis, and other tumors. An additional limitation of MRI is inconsistent ability to identifying the nidus. Radiographs showing an intracortical nidus, which can display variable amounts of mineralization, surrounded by cortical thickening and dense sclerosis assists in the diagnosis [26]. This may be better appreciated on CT.

Chondroblastoma

**Figure 13:**
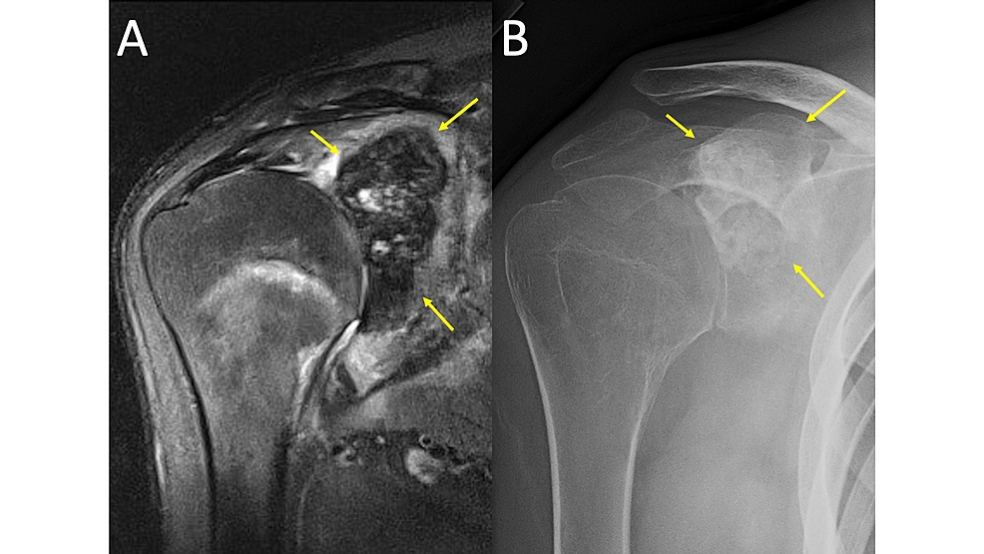
Chondroblastoma MRI coronal T2 fat saturation (FS) shoulder (13A) demonstrates a mildly heterogeneous, predominately hypointense, lobulated mass within the glenoid (yellow arrows). There is glenohumeral joint effusion, synovitis, and surrounding soft tissue and bone marrow edema. The imaging appearance suggests an aggressive process. A Grashey view radiograph of the right shoulder (13B) demonstrates a lobulated, sclerotic mass (yellow arrows) with an “arc and ring” appearance. Incidentally, there is severe glenohumeral joint space loss new from comparison radiographs (not shown) likely related to chronic inflammation of the glenohumeral joint. The location of the lesion and radiographic appearance is most consistent with chondroblastoma. The radiographic appearance is less aggressive than MRI findings.

Chondroblastoma is a benign bone tumor with a nonspecific clinical presentation of joint pain, tenderness to palpation, and soft tissue swelling in young patients generally less than 20 years of age. It frequently appears aggressive on MRI with periosteal reaction, extensive bone marrow edema, surrounding soft-tissue edema, joint effusion, and synovitis (Figure 13, panel A). Radiographs show a less aggressive appearing geographic lucent lesion with variable amounts of the ring and arc mineralized chondroid matrix and sclerotic margins located eccentrically within the epiphysis (Figure 13, panel B). The extent of mineralization of the chondroid matrix can increase over time, a feature that can also be seen with enchondromas.

Paget Disease

**Figure 14:**
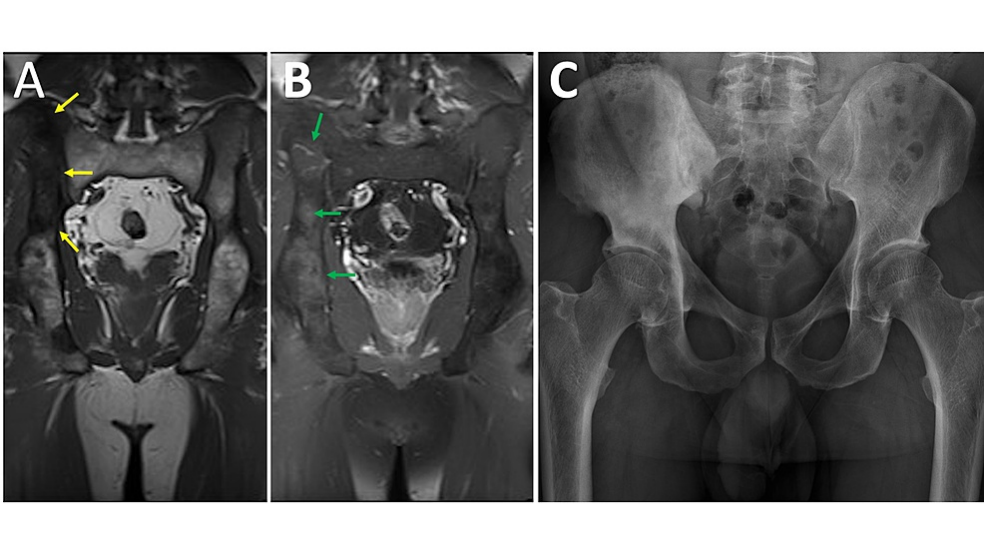
Paget MRI coronal T1 of the pelvis (14A) and coronal T1 post-contrast fat saturation (FS) of the pelvis (14B) demonstrates T1 hypointensity (yellow arrows) in the cranial aspect of the right iliac wing with diffuse speckled contrast enhancement (green arrows) relative to the contralateral osseous structures. A frontal radiograph of the pelvis (14C) demonstrates hypertrophic right side iliac wing and innominate bone with a slightly expanded appearance and thickened trabecula. No evidence of periarticular soft tissue calcification, destructive or blastic lesion, or other abnormality.

Paget disease is usually asymptomatic for years and can present with a variety of signs/symptoms, including bone pain, osseous deformity, hearing loss, neurological issues from spinal stenosis, warm skin, or fracture. MRI findings are variable and depend on the location and stage of the disease; early destructive stage (primarily lytic), intermediate stage (mixed lytic and blastic), and late inactive stage (primarily blastic). A long-standing disease has a dominant signal intensity similar to fat and is seen with most patients. The intermediate stage can show low T1 signal with high T2 signal and enhancement, all of which are likely related to the presence of granulation tissue, hypervascularity, and edema of active disease (Figure 14, panels A-B). The low T1/T2 signal seen in late disease, secondary to compact bone or fibrous tissue formation, is the least common presentation [27]. The variable, heterogeneous, infiltrative appearance of the marrow can be mistaken for an infiltrative marrow process such as myeloma, leukemia, infection, or metastasis.

The pathognomonic findings of trabecular and cortical thickening with enlargement of the bone, which are easily recognized on radiographs are often overlooked or misinterpreted on MRI (Figure 14C). Other classic radiographic imaging features such as osteoporosis circumscripta, cotton wool skull, widened diploic space, picture frame sign of the vertebral body, and bowing deformities of the long bones may also be missed on MRI because they have been traditionally taught and presented on radiographs. It is important to be able to recognize classic imaging findings across multiple imaging modalities.

## Conclusions

Radiographs have been shown to be helpful in the interpretation of MRI examinations in the majority of cases, especially when evaluating the aggressiveness of a lesion, the osseous anatomy or distribution of disease, and calcifications and gas in soft tissue. There are numerous processes to include various arthropathies, infection, trauma, and neoplasm, which may have nonspecific, easily overlooked, and/or confusing features on musculoskeletal MRI examinations. Comparison radiographs, when available, can more clearly delineate findings that may be subtle on MRI allowing for a more specific differential diagnosis. This decreases the likelihood of further unnecessary workup. Knowledge of these entities and their sometimes subtle or confusing MRI appearance is essential to the successful interpretation of musculoskeletal MRI.
